# Improving Retention of Very Old Participants in Longitudinal Research: Experiences from the Newcastle 85+ Study

**DOI:** 10.1371/journal.pone.0108370

**Published:** 2014-10-10

**Authors:** Karen Davies, Andrew Kingston, Louise Robinson, Joan Hughes, Judith M. Hunt, Sally A. H. Barker, June Edwards, Joanna Collerton, Carol Jagger, Thomas B. L. Kirkwood

**Affiliations:** 1 Newcastle University Institute for Ageing, Campus for Ageing and Vitality, Newcastle upon Tyne, United Kingdom; 2 Institute of Health and Society, Newcastle University, Newcastle upon Tyne, United Kingdom; 3 Institute for Cell and Molecular Biosciences, Newcastle University, Newcastle upon Tyne, United Kingdom; Karolinska Institutet, Italy

## Abstract

**Background:**

People aged 85 and over are often excluded from research on the grounds of being difficult to recruit and problematic to retain. The Newcastle 85+ study successfully recruited a cohort of 854 85-year-olds to detailed health assessment at baseline and followed them up over 3 phases spanning 5 years. This paper describes the effectiveness of its retention strategies.

**Methods:**

Primary retention strategies involved meticulous management of contact information and active maintenance of contact with participants between research visits and between phases of the study. For statistical analysis, data on post-inclusion attrition over the 3 follow-up phases was separated into ‘death’ and ‘withdrawal’ categories, with sub-categories ‘health’ and ‘non-health’ reasons created for ‘withdrawal’. Multinomial logistic regression was used to determine if particular socio-demographic and health characteristics were associated with post-inclusion attrition due to withdrawal at each of the 3 phase-to-phase transition points.

**Results:**

For both sexes, at successive follow-up phases there was a decrease in attrition due to withdrawal and an increase due to death. Withdrawal was most prevalent between baseline and phase 2. Across the 5 years of the study total post-inclusion (post-baseline) attrition due to death accounted for a 40% (344/854) loss to cohort and total post-inclusion attrition due to withdraw a 19% (166/854) loss to cohort, with health reasons for withdrawal becoming more dominant over time. Adjusting for sex, parsimonious modelling showed only occupational class (National Statistics Socio-economic Classification) to be associated with withdrawal and only between baseline and phase 2 (routine/manual compared to managerial (OR 3.41; 95% CI [1.23 to 9.44]).

**Conclusion:**

Following successful recruitment, we retained a high proportion of participants from a very old age group over 5 years of longitudinal research. No strong predictors of post-inclusion attrition due to withdrawal were found, suggesting the general effectiveness of our retention strategies.

## Introduction

People aged 85 years and older, the ‘very old’, are the fastest growing age-sector of the population in many countries. They are also assumed to be the most demanding of care [1], with little in the way of direct evidence.

A small number of longitudinal studies focussing on the actual needs and health-seeking behaviours of the very old are emerging, these include the Leiden 85+ Study [2], the Danish nonagenarian and centenarian studies [3], the Life and Living in Advanced Age Study in New Zealand (LiLACS NZ) [4], and in the UK the Medical Research Council Cognitive Function and Ageing Study (MRC CFAS) [5], the English Longitudinal Study of Ageing (ELSA) [6] and the Newcastle 85+ Study [7]. Nevertheless, very old people remain as seriously under-represented in research studies, despite reports reviewing ways to improve recruitment of older people to research through good practice [8], [9]. Once recruited to research, a related issue is how to *retain* very old participants, particularly across successive phases of longitudinal studies, where the risk of dropout threatens the validity, generalizability and cost-effectiveness of research [10]–[16]. The Newcastle 85+ Study, a population-based longitudinal study of health in the over-85s, achieved a high level of initial recruitment at baseline [9]. We here describe the effectiveness of retention strategies employed in the study's three follow-up phases spanning five years.

## Methods

### Ethics

Initial approval was given by Newcastle and North Tyneside Local Research Ethics Committee 1 on the 24^th^ February 2006. The REC reference number: The Newcastle 85+ Study: Biological, Clinical and Psychosocial factors associated with healthy ageing is 06/Q0905/2. Written informed consent was obtained from participants; where individuals lacked capacity to consent, for example because of cognitive impairment, a formal written opinion was sought from a relative or carer [9].

### Conduct of the study

**Figure 1:**
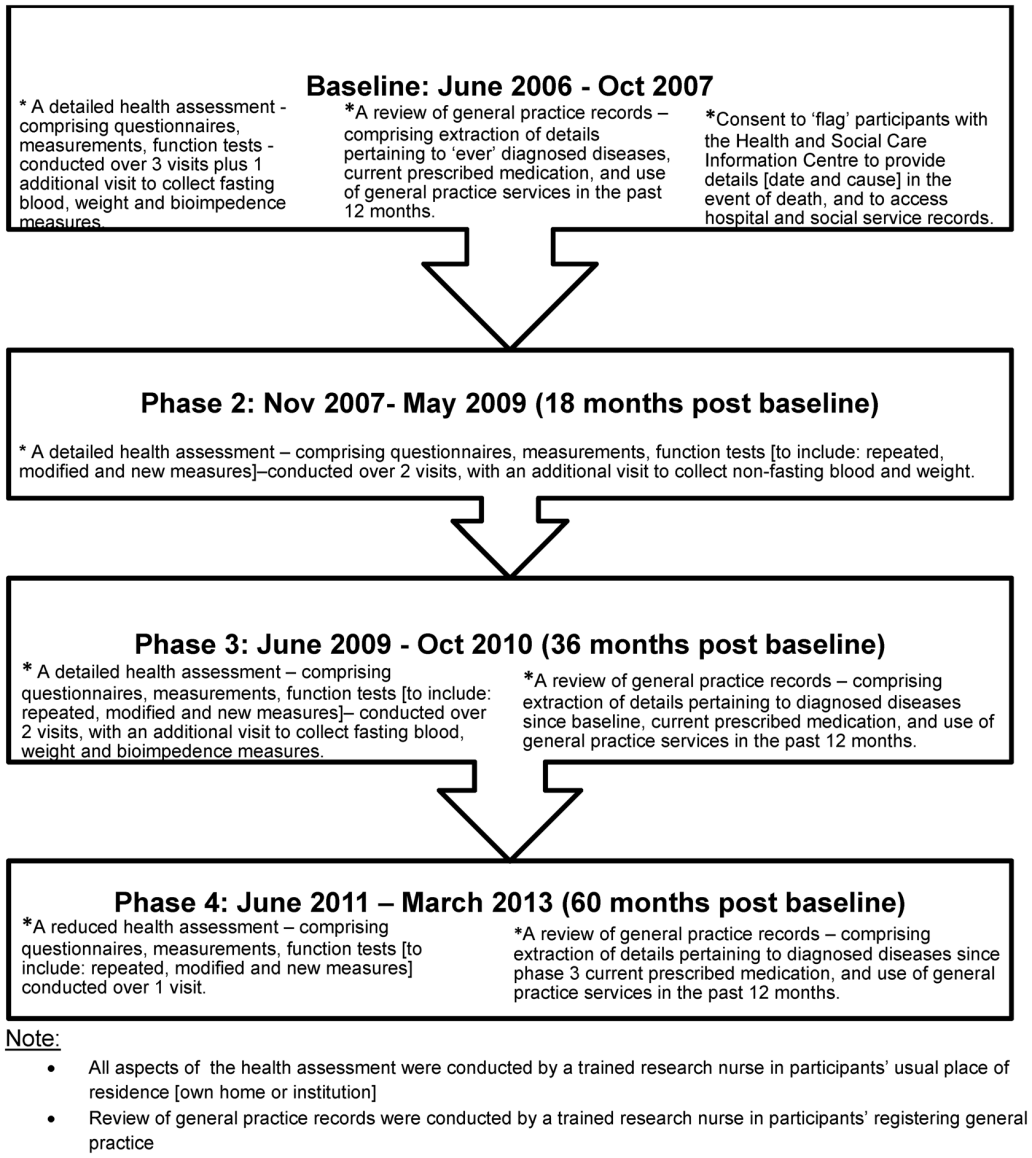
The Newcastle 85+ Study Timeline and Overview. A diagrammatic representation showing overall timescale of the Newcastle 85+ study and detailing content for each phase of the study.

The Newcastle 85+ Study approached all people turning 85 in 2006 and permanently registered with participating general practices (83% 53/64) in Newcastle or North Tyneside. Participating practices were similar to those which did not participate across key practice variables [7]. Individuals consented to health assessments (HA) and/or a review of their general practice medical records (GPRR). HA was conducted at baseline and then at 3 follow-up phases (at 18 months [Phase 2], 3 years [Phase 3] and 5 years [Phase 4]) by a research nurse in the participant's usual residence. GPRR was conducted at baseline, and at phases 3 and 4, in order to obtain information on disease status, medication and use of general practice GP services (Figure 1).

### Attrition Data

Reasons for non-participation at baseline and post inclusion (post-baseline) attrition -due to death and -due to withdrawal were recorded, with the latter being sub-categorised into two groups: i) health - the participant's own health status was given as a major factor, and ii) non-health (included ‘no reason given’ as participants were advised they did not have to provide a reason).

### Retention Strategies

Maintaining contact and managing up-to-date contact information plays a vital role in participant retention [11], [17]. Information such as time of day, day of week, and date of any contact attempt, even if failed, was fully recorded alongside address, telephone number, and consultee or family representative details. Participants were asked to nominate an alternative contact person in case their health should deteriorate. Salient points and outcomes of conversations with participants or their families/carers were also recorded, including caring responsibilities of the participant, family dynamics and health, planned holidays and appointments, significant relationships with neighbours and key holders.

The nurse team used such information to assess whether participants, or where appropriate their family/carers, might benefit from additional brief personal visits, usually whilst the research nurse was in the area or visiting another participant in a particular care home. Balancing the maintenance of contact, as opposed to causing annoyance or distress, was founded on our actively managed awareness of participants' individual circumstances and their changing needs. All such details were held securely on an electronic database (Microsoft Access), and regularly updated and crosschecked, with the nurse team taking ownership of this task.

Additionally, strategies to keep the study ‘alive’ for participants, both between research visits within a phase and between research phases were employed, including actions recommended by others [11], [17]–[19] such as reminder letters, newsletters, telephone calls, Christmas cards, and gifts of everyday items for instance a mug and tea-towel, all displaying the study logo.

The most ambitious efforts to maintain contact and keep the study ‘alive’ included holding a celebratory event for participants and their families during phase 3 of the study. Lunch and refreshments were provided, along with entertainment from a drama group and a choir. Transport to and from the venue was made available to those who needed this. This event provided an opportunity to share preliminary research findings and for open discussion between participants and the study team. Also, the publication of a book telling the ‘story’ of the Newcastle 85+ study from participant and research team perspectives; how the study came into being, what had been discovered so far (including ‘lay’ synopsis of publications), and why it mattered [20]. A copy of the book was given to each participant along with a signed letter of thanks. In the event of participant death books were given to significant others.

### Statistical methods

To determine if particular socio-demographic and health characteristics were associated with post-inclusion attrition due to withdrawal, multinomial logistic regression was used to compare three groups across all the phase-to-phase transition points: those who withdrew for health reasons, those who withdrew for non-health reasons, and those who remained in the study. The same socio-demographic and health characteristics - years of education, National Statistics Socio-economic Classification (NS-SEC [21]), area deprivation (Index of Multiple Deprivation IMD [22]), marital status, living arrangements, housing type, self-rated health (SRH), depression, disability, cognition, disease burden (disease count – Table 1. Box 1), and health service use (comprising: GP consultations and hospital inpatient in past year) - were examined at each analysis point; these were time-varying if measured at subsequent phases and time-constant otherwise. These characteristics were selected upon existing evidence linking them to post-inclusion attrition due to withdrawal in longitudinal cohorts of older adults [23]–[30]. Initially analysing variables individually, then constructing a parsimonious model using all variables by means of a back step approach. No models were retained in the final longitudinal model (baseline to phase 4). We therefore repeated analysis for each phase-to-phase transition separately. Models were also adjusted for sex since others have shown this to impact on attrition. [31], [32] and we have already shown that very old women have significantly greater morbidity and disability [7]. Analyses were carried out in Stata 12.1 (StataCorp. 2011. *Stata Statistical Software: Release 12*. College Station, TX: StataCorp LP).

**Table 1:** Box 1: Disease Count.

DISEASE GROUP	ASCERTAINMENT CRITERIA
Arthritis*	Any recorded diagnosis of Generalised Osteoarthritis, Hand, Hip and Knee Osteoarthritis Rheumatoid, Degenerative, Poly, Gouty, Septic, Peri, Lumbar Spondylosis, Cervical Spondylosis, Ankylosing Spondylitis and Psoriatic Arthropathy
Hypertension*	Any recorded diagnosis of Hypertension
Cardiac disease*	Heart Failure, Ischaemic heart disease (Angina, Myocardial Infarction, Coronary Artery Bypass Graft, Coronary Angioplasty/Stent)
Respiratory disease*	Bronchiectasis, Pulmonary Fibrosis, Fibrosing Alveolitis, Asbestosis, Pneumoconiosis, Asthma, Chronic Bronchitis, Emphysema, COPD
Cerebrovascular disease*	Stroke, Transient Ischaemic Attack, Carotid Endarterectomy
Diabetes mellitus*	Type I, Type II and type unspecified
Cancer*	Any cancer diagnosis in past 5 years excluding non-melanoma skin cancer
Cognitive Impairment^†^	Standardised Mini-Mental State Examination (sMMSE) score of ≤21

NOTE:

• * Data taken from GP record review.

• † Score calculated from health assessment (sMMSE).

## Results

### Baseline recruitment

Contact was established with 97% (1409/1459) of those invited to participate in the study and written informed consent was obtained from 74% (1042/1409) of these [9]. 851 individuals consented to undergo both HA and GPRR; 3 gave consent to HA only (declining GPRR); 188 consented to GPRR only (declining multidimensional health assessment); and 358 declined all participation. Women were more likely than men to decline either all participation or participation in HA, however the resulting 85+ cohort (those participating in HA n854) was socio-demographically representative of the local population, and of England and Wales, including the proportion in care homes [7].

### Retention

**Figure 2:**
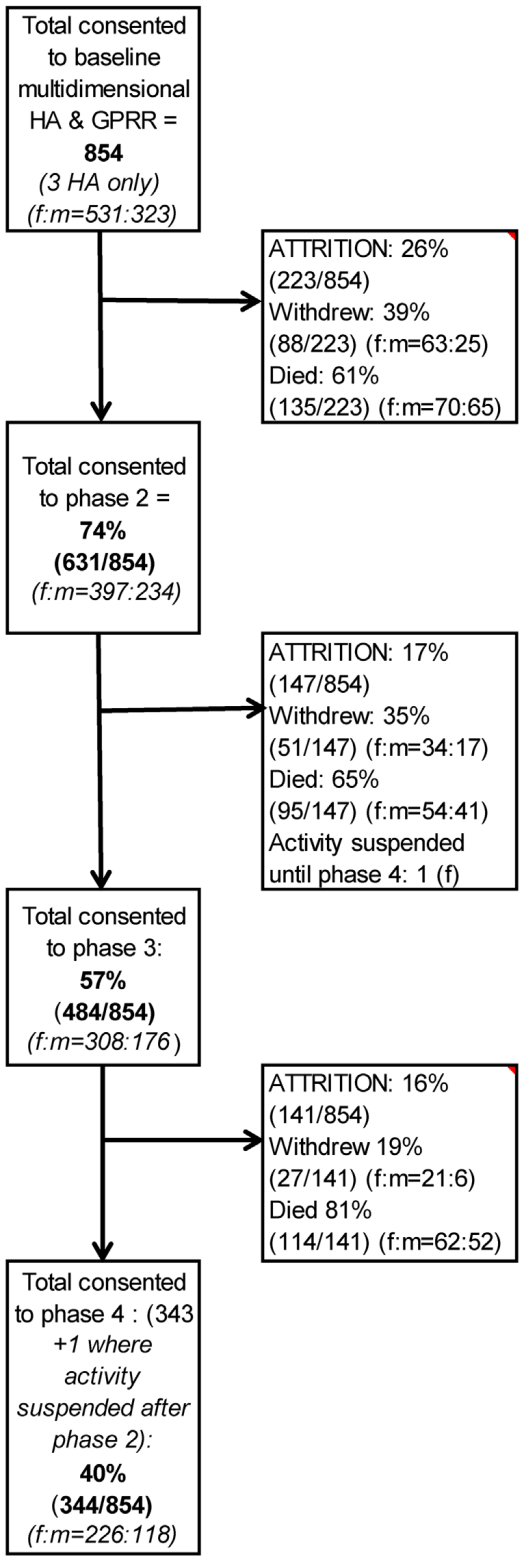
The Newcastle 85+ Study cohort phase to phase retention profile. A diagrammatic representation of activity for the Newcastle 85+ study cohort (n854). Detailing retention of the cohort at each phase, showing figures for men and women separately, and attrition due to withdrawal and attrition due to death separately.

Figure 2 presents the phase-to-phase retention profile for the 85+ cohort illustrating overall retention rates of 74% (631/854) baseline to phase 2 (attrition -due to death 16% (135/854), –due to withdraw 10% (88/854)), 57% (484/854) phase 2 to phase 3 (attrition -due to death 11% (95/854), –due to withdraw 6% (51/854)), and 40% (344/854) phase 3 to phase 4 (attrition -due to death 13% (114/854), –due to withdraw 3% (27/854)). Ultimately across the five years of the study 40% of participants (344/854) were lost to death. Of the survivors, two-thirds were retained (344/510 (or 40% (344/854) of 85+ cohort)) and one-third lost to withdrawal (166/510 (or 19% (166/854) of 85+ cohort)).

### i) Sex and Post-inclusion attrition

Figure 2 also presents results for men and women separately, where for both sexes at successive follow-up phases, we observed a *decrease* in post-inclusion attrition due to withdrawal and an *increase* due to death. Slightly more women than men withdrew at each follow-up phase ((baseline to phase 2 f:m = 12%∶8%); (phase 2 to phase 3 f:m = 9%∶7%) and (phase 3 to phase 4 f:m = 7%∶3%)) but fewer women died ((baseline to phase 1 f:m = 13%∶20%); (phase 2 to phase 3 f:m = 14%∶18%) and (phase 3 to phase 4 f:m = 20%∶30%)), though gender differences were not significant (p = 0.1325).

### ii) Reasons for post-inclusion attrition due to withdrawal

Table 2 presents the reasons given by the 85+ cohort (for men and women separately), or their families where indicated, for withdrawal at each follow-up phase. At transition from baseline to phase 2, health reasons accounted for 40% (35/88) of all withdrawals with ‘too ill’ being the most frequently reported reason for both men and women (83%; 29/35). For non-health reasons, accounting for 60% (53/88) of all withdrawals, ‘too much hassle’ was most frequently reported (28%; 15/53), driven by women (f:m = 13∶2). At transition from phase 2 to phase 3, health reasons increased to account for 69% of all withdrawals (35/51) with ‘too ill’ again the most frequent reason for both sexes (83%; 29/35). Non-health reasons reduced considerably to account for 31% (16/51) of all withdrawals, again driven by women (f:m = 14∶2). Of the non-health category, ‘no reason’ was the most frequently recorded response (38%; 6/16), with a f:m ratio of 5∶1, and ‘lost interest’ a close second (31%; 5/16) populated entirely by women. At transition from phase 3 to phase 4, although the proportion of withdrawals due to health reasons decreased, they still accounted for the majority of withdrawals (56%; 15/27), with ‘too ill’ being the most frequently reported (52%; 14/15), and led by women (f:m = 13∶1). Non-health reasons accounted for 44% of all withdrawals (12/27), with ‘no reason’ most frequently reported (58%; 7/12), led by women (f:m = 5∶2).

**Table 2:** Reasons for withdrawal.

	REASON FOR WITHDRAW	BASELINE TO PHASE 2 AS % (OF HEALTH n35 OR NON-HEALTH n53)	PHASE 2 TO PHASE 3 AS % (OF HEALTH n35 OR NON-HEALTH n16)	PHASE 3 TO PHASE 4 AS % (OF HEALTH n15 OR NON-HEALTH n12)
HEALTH	**Too ill**	**83% (29/35)**	**83% (29/35)**	**93% (14/15)**
	*Male (simple count)*	9	13	1
	*Female (simple count)*	18	16	13
	**Too tired**	**9% (3/35)**	**9% (3/35)**	**-**
	*Male*	-	-	-
	*Female*	3	3	-
	**Withdrawn by family** (lost capacity)	**6% (2/35)**	**3% (1/35)**	**-**
	*Male*	1	-	-
	*Female*	1	1	-
	**Too many other health appts.**	**3% (1/35)**	**6% (2/35)**	**7% (1/15)**
	*Male*	-	2	-
	*Female*	1	-	1
	***Total % health***	***40% (35/88)***	***68% (35/51)***	***56% (15/27)***
NON-HEALTH	**Concerns about privacy**	**11% (6/53)**	**-**	**-**
	* Male*	2	-	-
	* Female*	4	-	-
	**Didn't enjoy last time**	**-**	**6% (1/16)**	**-**
	* Male*	-	-	-
	* Female*	-	1	-
	**Lost interest**	**23% (12/53)**	**6% (5/16)**	**-**
	* Male*	1	-	-
	* Female*	11	5	-
	**Lost to follow up** (moved out of area)	**4% (2/53)**	**-**	**25% (3/12)**
	* Male*	2	-	2
	* Female*	-	-	1
	**Only ever agreed to one interview**	**2% (1/53)**	**-**	**-**
	* Male*	1	-	-
	* Female*	-	-	-
	**Recent bereavement**	**2% (1/53)**	**13% (2/16)**	**-**
	* Male*	-	1	-
	* Female*	1	1	-
	**Spouse unwell**	**-**	**6% (1/16)**	**8% (1/12)**
	* Male*	-	-	1
	* Female*	-	1	-
	**Too busy**	**4% (2/53)**	**6% (1/16)**	**-**
	* Male*	2	-	-
	* Female*	-	1	-
	**Too much hassle**	**28% (15/53)**	**-**	**8% (1/12)**
	* Male*	2	-	-
	* Female*	13	-	1
	**No reason given**	**23% (12/53)**	**38% (6/16)**	**58% (7/12**
	* Male*	5	1	2
	* Female*	7	5	5
	**Destroy all**	**4% (2/53)**	**-**	**-**
	***Total % non-health***	***60% (53/88)***	***31% (16/51)***	***44% (12/27)***

NOTE:

• Individual % are rounded and as such may not total 100%.

### iii) Socio-demographic and health characteristics associated with post-inclusion attrition due to withdrawal

Investigation of attrition between baseline and phase 2 highlighted associations with three of the socio-demographic and health characteristics examined (Table 3). Participants who belonged to the ‘withdrew for health reasons’ group versus those who continued participation were nearly five times as likely to have held routine/manual occupations during their working life (OR: 5.38; 95% CI [1.59 to 18.19]). A similar pattern was seen for those who had rated their health as fair or poor at baseline (OR: 3.09; 95% CI [1.15 to 8.31]). However, confidence intervals surrounding these odds ratios are wide due to the small numbers of people belonging to these groups. Participants were 51% less likely to have withdrawn for health reasons versus continued to participate if they had difficulty with 1 to 6 activities of daily living ((I)ADL's) compared with those who had difficulty with 7 or more activities (OR: 0.49; 95% CI (0.27 to 0.91)). When examining attrition between phases 2 and 3, participants withdrawing for non-health reasons were more likely to live in areas with higher deprivation (IMD) or in ‘institutional care’ housing type when compared with those continuing participation. However, numbers were low in these groups giving wide margins in the calculated confidence intervals. When examining attrition between phases 3 and 4, those who suffered from severe depression were more likely to withdraw for health reasons than those continuing participation; again a wide confidence interval prevents drawing any definitive conclusion. Constructing a parsimonious model yielded results indicating that only NS-SEC showed an association with post-inclusion attrition between baseline and phase 2 after adjusting for sex, with participants belonging to the ‘withdrew for health reasons’ group being significantly more likely to have held routine/manual occupations (OR 3.41; 95% CI [1.23 to 9.44]) than those who remained in the study. There were no detectable associations with participation status at the phase 2 to 3 or phase 3 to 4 transition points when all variables were considered together.

**Table 3:** Key health and socio-economic characteristics associated with phase to phase attrition.

		BASELINE TO PHASE 2		PHASES 2 TO 3		PHASES 3 TO 4	
		Withdraw for Non-Health Reasons	Withdraw for Health Reasons	Withdraw for Non-Health Reasons	Withdraw for Health Reasons	Withdraw for Non-Health Reasons	Withdraw for Health Reasons
Men		**Referent**	**Referent**	**Referent**	**Referent**	**Referent**	**Referent**
Women		**1.51 (0.82–2.75)**	**1.31 (0.59–2.92)**	**2.57 (0.86–7.72)**	**0.70 (0.33–1.50)**	**0.84 (0.27–2.62)**	**6.82 (0.88–52.75)**
Years in Education:							
	**12+**	**Referent**	**Referent**	**Referent**	**Referent**	**Referent**	**Referent**
	**10–11**	**0.96 (0.36–2.56)**	**1.52 (0.29–8.01)**	**0.81 (0.21–3.13)**	**1.74 (0.45–6.77)**	**0.19 (0.02–1.73)**	**2.25 (0.23–22.23)**
	**0–9**	**1.14 (0.49–2.64)**	**2.32 (0.53–10.07)**	**0.80 (0.25–2.52)**	**1.39 (0.40–4.88)**	**0.50 (0.15–1.72)**	**2.50 (0.31–19.95)**
NS-SEC (3 class version: based upon **head of household's main occupation)							
	**Managerial/Professional**	**Referent**	**Referent**	**Referent**	**Referent**	**Referent**	**Referent**
	**Intermediate**	**2.21 (0.92–5.31)**	**2.65 (0.52–13.40)**	**0.36 (0.04–2.97)**	**0.84 (0.22–3.18)**	**1.41 (0.33–6.12)**	**0.94 (0.18–5.01)**
	**Routine/Manual**	**1.89 (0.95–3.77)**	**5.38 (1.59–18.19) ‡**	**1.21 (0.46–3.20)**	**1.37 (0.59–3.18)**	**0.92 (0.26–3.26)**	**1.29 (0.40–4.17)**
Area Deprivation (calculated index of multiple deprivation IMD):							
	**0–25**	**Referent**	**Referent**	**Referent**	**Referent**	**Referent**	**Referent**
	**25–75**	**1.57 (0.75–3.29)**	**0.81 (0.32–2.02)**	**8.19 (1.06–62.93) ‡**	**0.97 (0.42–2.29)**	**1.41 (0.27–7.40)**	**0.32 (0.09–1.13)**
	**75–100**	**2.04 (0.91–4.56)**	**1.28 (0.48–3.39)**	**8.78 (1.06–72.45) ‡**	**0.70 (0.23–2.14)**	**3.93 (0.77–20.04)**	**0.56 (0.14–2.25)**
Self-Rated Health (compared to others of same age):							
	**Excellent/Very Good**	**Referent**	**Referent**	**Referent**	**Referent**	**Referent**	**Referent**
	**Good**	**1.32 (0.70–2.50)**	**1.96 (0.76–5.05)**	**0.66 (0.22–1.97)**	**0.77 (0.30–2.00)**	**1.50 (0.39–5.71)**	**1.44 (0.43–4.84)**
	**Fair/Poor**	**1.59 (0.77–3.28)**	**3.09 (1.15–8.31) ‡**	**1.18 (0.39–3.54)**	**1.77 (0.72–4.33)**	**2.48 (0.60–10.25)**	**0.99 (0.19–5.26)**
Marital Status:							
	**Married**	**Referent**	**Referent**	**Referent**	**Referent**	**Referent**	**Referent**
	**Widowed**	**1.18 (0.63–2.21)**	**0.66 (0.30–1.42)**	**1.11 (0.39–3.18)**	**1.01 (0.43–2.37)**	**1.02 (0.26–3.93)**	**1.15 (0.30–4.33)**
	**Never/Divorced/Separated**	**0.98 (0.36–2.61)**	**(no obs)**	**1.86 (0.48–7.20)**	**0.29 (0.04–2.38)**	**1.51 (0.24–9.44)**	**1.51 (0.24–9.44)**
Living Arrangements*:							
	**Alone**	**Referent**	**Referent**	**Referent**	**Referent**	**Referent**	**Referent**
	**With spouse**	**1.24 (0.45–3.41)**	**1.01 (0.26–3.92)**	**2.31 (0.55–9.60)**	**0.29 (0.04–2.37)**	**0.59 (0.06–5.86)**	**0.88 (0.08–10.07)**
	**With others**	**1.03 (0.52–2.02)**	**0.81 (0.34–1.95)**	**0.97 (0.30–3.17)**	**0.93 (0.39–2.17)**	**0.77 (0.19–3.08)**	**1.49 (0.31–7.08)**
Housing type:							
	**Standard**	**Referent**	**Referent**	**Referent**	**Referent**	**Referent**	**Referent**
	**Sheltered**	**1.54 (0.74–3.20)**	**1.07 (0.36–3.17)**	**0.88 (0.25–3.10)**	**0.36 (0.08–1.56)**	**(no obs)**	**1.54 (0.40–5.85)**
	**Institutional**	**2.42 (1.01–5.77)**	**1.20 (0.27–5.29)**	**4.53 (1.20–17.12) ‡**	**(no obs)**	**4.12 (0.82–20.70)**	**5.04 (0.98–25.91)**
Depression (GDS -15 score) ^†^:							
	**None**	**Referent**	**Referent**	**-**	**-**	**Referent**	**Referent**
	**Mild**	**0.42 (0.10–1.79)**	**2.17 (0.77–6.11)**	**0.66 (0.15–2.93)**	**0.66 (0.15–2.93)**	**0.64 (0.08–5.13)**	**3.04 (0.75–12.24)**
	**Severe**	**-**	**1.31 (0.29–5.88)**	**1.46 (0.32–6.63)**	**2.92 (0.92–9.25)**	**(no obs)**	**7.21 (1.70–30.52) ‡**
Disability (I)ADL's score^◊^:							
	**7+**	**Referent**	**Referent**	**Referent**	**Referent**	**Referent**	**Referent**
	**1–6**	**0.49 (0.27–0.91)‡**	**0.64 (0.30–1.38)**	**0.52 (0.20–1.34)**	**0.62 (0.27–1.42)**	**0.44 (0.13–1.56)**	**0.71 (0.23–2.22)**
	**None**	**0.55 (0.26–1.17)**	**(no obs)**	**0.73 (0.19–2.86)**	**0.59 (0.16–2.20)**	**1.99 (0.45–8.82)**	**0.66 (0.07–5.90)**
Cognitively Impaired (sMMSE< = 21)		**1.64 (0.74–3.69)**	**1.64 (0.55–4.91)**	**Not completed**	**Not completed**	**2.27 (0.60–8.61)**	**(too few obs)**
Hospital Inpatient in past 12 months		**0.87 (0.43–1.77)**	**1.11 (0.44–2.80)**	**0.42 (0.12–1.45)**	**0.89 (0.35–2.30)**	**0.62 (0.13–2.84)**	**1.51 (0.45–5.02)**
Disease count^¥^		**0.95 (0.76–1.19)**	**1.15 (0.86–1.54)**	**Not completed**	**Not completed**	**0.76 (0.45–1.29)**	**0.98 (0.61–1.59)**
Consultations with GP in past 12 months		**0.97 (0.91–1.03)**	**1.02 (0.96–1.10)**	**Not completed**	**Not completed**	**0.99 (0.89–1.10)**	**1.02 (0.95–1.10)**

NOTE:

• All data taken from HA excluding 'Disease count' and 'Consultations' taken from GPRR.

• NS-SEC =  National Statistics Socio-economic Classification (3 class version: based upon head of household main occupation – **using participant's (or in the case of married women the husband's) main occupation during their working life; GDS =  geriatric depression scale; sMMSE  =  standardised mini-mental state examination.

• *Excludes participants in institutional care.

• †GDS omitted if score <15 on sMMSE.

• ◊No of activities of daily living (I)ADL's) carried out with difficulty or requiring an aid, appliance, or personal help.

• ¥Disease count derived from 8 chronic diseases from GPRR (**Table 3** **. Box 1**).

• ‡Statistically significant.

## Discussion

Whilst the greatest attrition in our study occurred between baseline and phase 2, overall retention rates were good and, although showing some associations, statistical analysis detected no strong predictors of attrition due to withdrawal, suggesting minimal attrition bias. While our findings accord with the fact that women live longer than men [33] but with greater levels of disability [34]–[37], they conflict with those reporting that post-inclusion attrition rates due to withdrawal increase with length of follow-up [13], [38]. A systematic review found that only increasing age and cognitive impairment were significantly associated with greater attrition due to withdrawal in longitudinal studies of ageing [28]. Although we could not assess the impact of age from our single birth cohort, we did not find evidence that attrition was higher in those with cognitive impairment. Our experience suggests some notion of increasing loyalty, or investment over time, by our participants as well as continued interviewer training. We attribute our findings that attrition did not appear to be related to socio-economic status or health to the success of our retention strategies.

The strategies of maintaining participant contact additional to scheduled study visits undoubtedly came with resource costs. However, whereas other studies, such as Blanton et al's [39] study of stroke survivors, reported that such strategies are too labour intensive and yield little benefit, our experience was that in longitudinal research focussing solely on a very old population, there was much to be gained.

### Challenges in maintaining contact

A particular challenge to maintaining participant contact was the use of a telephone system withholding caller ID, as does the Newcastle University system. This problem was detected when participants, first labelled as ‘unable to establish telephone contact’ and then successfully contacted by alternative approaches, disclosed that they did not answer the telephone, or had been instructed by their family not to do so, if caller ID was withheld. To resolve this, in addition to contact protocols stipulating that telephone calls be made on alternate days and at different times of the day [9], the nurse team attempted at least one or two calls using a mobile phone.

Moving home is commonplace among older populations, for example to live in closer proximity to family or to move into a care home [40]. On average 6% of the Newcastle 85+ Study cohort changed address at each of the 3 follow-up phases (40;49;57/854), with over 50% of these being ‘new’ transitions into care homes from private homes. Clearly, change of address is a challenging issue for researchers attempting to remain in contact with study participants. Other longitudinal studies involving older people report change of address as a major contributory factor of loss to follow-up accounting for 9% of total attrition over 4 years [27]. Across the duration of our study, only 0.5% (5/854) of participants were lost to follow up. This was made possible through effective engagement of various ‘gatekeepers’, often in combination. Researchers are urged to use great sensitivity when participants move into care, as these transitions are often associated with deterioration in health and independence. In such situations, family emotions of anxiety and guilt are not uncommon and notifying researchers of a change of address is not a priority. Maintaining a good relationship with general practice (GP) staff and, where relevant, care home staff proved valuable, as they were supportive in forwarding study contact letters asking participants or relatives to contact research staff. A further resource in re-establishing contact with participants who changed address existed for those who had consented to having their records flagged with the Health and Social Care Information Centre. This enabled us to identify and contact a new registering general practice, again to request the forwarding of a study contact letter. If the new practice was not already participating in, or indeed aware of, the study, we reverted to our GP recruitment protocol [9], guiding surgeries through the aims of the study.

### Using participant feedback to overcome barriers

Feedback not only relates to providing participants with ongoing information but also to the collection of information from the participants' perspectives. Where given, we formally captured reasons for withdrawal, as well as the experiences of participants who continued with the study, asking at the end of each health assessment ‘How did you find this interview?’, and recording responses verbatim.

Effective use was made of this information through ‘reflexive practice’ techniques at scheduled weekly meetings of the research nurse team. This involved questioning our own attitudes, thoughts and reactions in relation to others and, if necessary, revising our ways of relating [41], [42]. Although participant withdrawal cannot be eliminated, this exercise increased the confidence of our research nurses in recognising and actively seeking to remove barriers to retention (Table 4).

**Table 4:** Potential Solutions to Post-inclusion Attrition Due to Withdraw.

REASONS FOR WITHDRAWAL	SOLUTIONS
No reason	*Prompt for more information using open/closed questions. When did ‘no reason’ most commonly occur? *Leave door open giving individual the opportunity to get back in touch
Too ill/Too tired	Timing: is the health problem transient? Explore if this is a new health event. Set time scale to ring back. Reduce research activity. Suspend rather than cease research activity.
Lost interested	Use knowledge to create interest. Ensure participant feels valued
Didn't enjoy last time	Try to determine if any specific aspect of assessment is causing problem and modify/reduce research activity to suit.
Too busy/Other appointments & commitments	Split visits into shorter duration. Flexibility: fit visits around the participant's ‘other’ commitments – offer ‘out of hours’ visits.
Visits too long	Try to pin down if any particular aspect of the assessment was as too much of a burden and modify to/reduce to suit. Check any previous technical problems that caused interview to be longer and reassure or modify to suit. Split visits into shorter duration. Take time out: Chat/break/take refreshment then assess if it is appropriate to re-engage the participant with the assessment. Be mindful of your own time constraints (other appointments). Acknowledge and focus on the positive of what has been achieved.
Lost to follow-up	Ensure all avenues of establishing whereabouts have been explored: review information held on database, alternate contacts, GP, care workers, trace service etc.

NOTE:

• *Solutions apply to each category.

### Motivating staff

In agreement with Patel et al [14] that research can be a solitary occupation, we found the continual motivation of staff to be crucial. EJiogu et al [43] offered specific recommendations such as staff incentives, including supporting pursuit of academic goals. We found this incentive successful not only in maintaining staff motivation but also in enhancing team knowledge. Other incentives included target-related bonus schemes, especially to compensate staff nearing the end of a fixed term appointment and postponing the search for alternative employment.

## Conclusions

It is imperative that participants have the right to withdraw from research at any time. However, added to those drivers aiming to “… get the right people into research, older people, people with comorbid diseases, and frail people. These are the people we care for …” [44] (p16), we consider it vitally important for researchers to examine reasons for attrition to ensure that attrition does not result from lack of understanding or barriers to participation which, if addressed through effective retention strategies, could protect each individual's right to continue their participation in research.
